# Intact sensory-motor network structure and function in far from onset premanifest Huntington’s disease

**DOI:** 10.1038/srep43841

**Published:** 2017-03-07

**Authors:** Martin Gorges, Hans-Peter Müller, Isabella Maria Sophie Mayer, Gesa Sophie Grupe, Thomas Kammer, Georg Grön, Jan Kassubek, G. Bernhard Landwehrmeyer, Robert Christian Wolf, Michael Orth

**Affiliations:** 1 Department of Neurology, University of Ulm, Ulm, Germany; 2 Section Neuropsychology and Functional Imaging, Department of Psychiatry, University of Ulm, Germany; 3 Center for Psychosocial Medicine, Department of General Psychiatry, Heidelberg University, Germany

## Abstract

Structural and functional changes attributable to the neurodegenerative process in Huntington’s disease (HD) may be evident in *HTT* CAG repeat expansion carriers before the clinical manifestations of HD. It remains unclear, though, how far from motor onset a consistent signature of the neurodegenerative process in HD can be detected. Twelve far from onset preHD and 22 age-matched healthy control participants underwent volumetric structural magnetic resonance imaging (MRI), diffusion tensor imaging (DTI), and resting-state functional MRI (11 preHD, 22 controls) as well as electrophysiological measurements (12 preHD, 13 controls). There were no significant differences in white matter macro- and microstructure between far from onset preHD participants and controls. Functional connectivity in a basal ganglia-thalamic and motor networks, all measures of the motor efferent and sensory afferent pathways as well as sensory-motor integration were also similar in far from onset preHD and controls. With the methods used in far from onset preHD sensory-motor neural macro- or micro-structure and brain function were similar to healthy controls. This suggests that any observable structural and functional change in preHD nearer to onset, or in manifest HD, at least using comparable techniques such as in this study, most likely reflects an ongoing neurodegenerative process.

In Huntington’s disease (HD), a monogenetic, autosomal dominant neurodegenerative disorder the CAG repeat expansion mutation in the Huntingtin (*HTT)* gene gives rise to a mutant huntingtin protein^1^. The mutant protein may confer a toxic gain of function. However, a loss of function of the normal huntingtin protein may also contribute to cause striatal and extra-striatal, e.g. cortical, neurodegeneration in HD. The neurodegenerative process in HD affects white and grey matter^2^, and eventually leads to the emergence of clinical signs, most commonly motor signs, that allow a clinical diagnosis of manifest HD. TRACK-HD, a large longitudinal study combining clinical and structural imaging data from both premanifest and early manifest HD participants, showed that clinical measures –e.g., motor and cognitive tasks performance–and volumetric imaging tracked disease evolution associated with manifest HD, i.e. after unequivocal motor signs of HD have been noted^3^. Structural differences in cerebral white and grey matter may be detectable as far as 15–20 years before onset of unequivocal signs of HD and have been reported using VBM^4^ and DTI^3^. Similar to structural abnormalities, cross-sectional task-based, or ‘resting-state’ functional MRI (rs-fMRI) studies have reported changes of brain function in preHD^5^,^6^. PREDICT-HD, a longitudinal study in premanifest HD (preHD), demonstrated that motor and cognitive task performance, and structural imaging (in particular putamen volume), improved predictions of motor diagnosis compared with models using *HTT* CAG repeat length and age^7^. This indicates that structural and functional changes attributable to the neurodegenerative process in HD may be evident before a clinical diagnosis of HD. It remains unclear, though, how far from motor onset a consistent signature of the neurodegenerative process in HD can be detected in preHD.

In a cohort of preHD participants very far from motor onset based on disease burden score^8^ and predictions of years to motor onset^9^, we used multimodal measurements to assess motor network integrity in terms of brain structure (VBM, DTI tractography), function (functional MRI and electrophysiology) and task performance. We focused on sensory-motor circuits because these circuits are relevant for the motor abnormalities that are an important part of the clinical phenotype in HD. In addition, macro-(volumetric) and microstructural (DTI) abnormalities have consistently been reported for the brain areas involved in sensory-motor circuits including cortical somatosensory projections, abnormal in manifest disease^10^, the primary sensory cortex, in which there is evidence of thinning prior to symptom onset^11^, and white matter changes assessed using DTI^11^. The reduction of somatosensory evoked potential (SEP) amplitudes of cortical components further suggests that brain function may also be abnormal^12^. We hypothesised that we would detect differences between controls and far from onset preHD participants in measure of brain function, i.e. functional MRI and electrophysiology, while we expected that measures of brain structure (VBM, DTI tractography) would be similar in the two groups.

## Results

### White and gray matter structure

**Figure 1:**
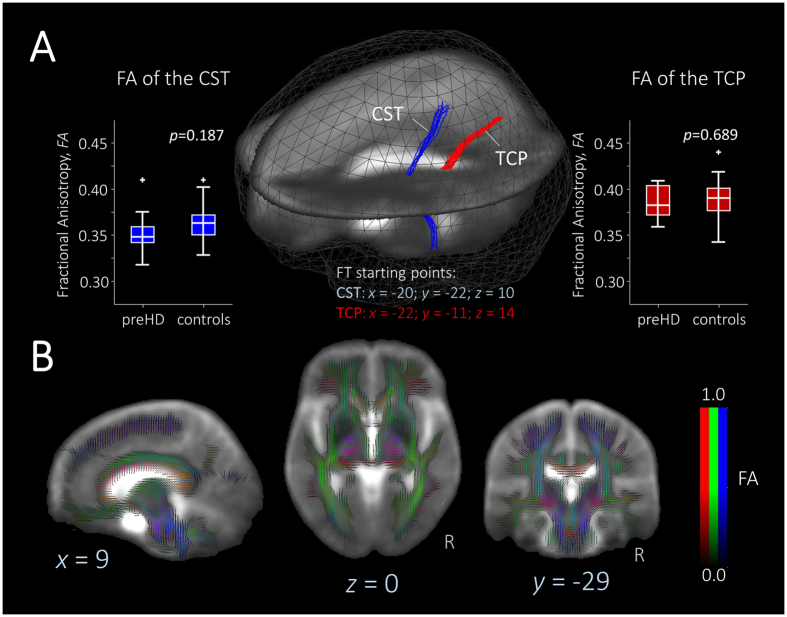
Diffusion tensor imaging tractography. **(A)** Fiber tracking in the cortico-spinal tract (**CST**, **blue**) and thalamo-cortical pathway (**TCP**, **red**) revealed no significant differences between far from onset preHD and controls in fractional anisotropy (FA) of the CST (boxplot left) or the TCP (boxplot, right). **(B)** Representative orthogonal slices in the MNI-space depict averaged FA-maps underlying fiber tracking.

We used DTI and whole-brain GMV measures to assess structural integrity. There were no significant differences in white matter micro- and macrostructure between far from onset preHD participants and controls. In particular, WBSS of FA-maps was similar in far from onset preHD participants and controls, i.e. voxel-wise group comparison showed no statistically significant differences. Fiber tracking of the CST and TCP showed no differences of FA values between far from onset preHD participants and controls (Fig. 1). In addition, AD-values for CST (*p* = 0.271) or for the TCP (*p* = 0.186) as well as RD-values for CST (*p* = 0.998) or for the TCP (*p* = 0.323) revealed no statistically significant differences. In whole-brain analyses, no significant differences between healthy controls and far from onset preHD were found. This was true for both GMV and WMV. In ROI-analyses, there were no significant caudate or putamen GMV differences between the groups.

### Similar functional brain maps in far from onset preHD and controls

**Figure 2:**
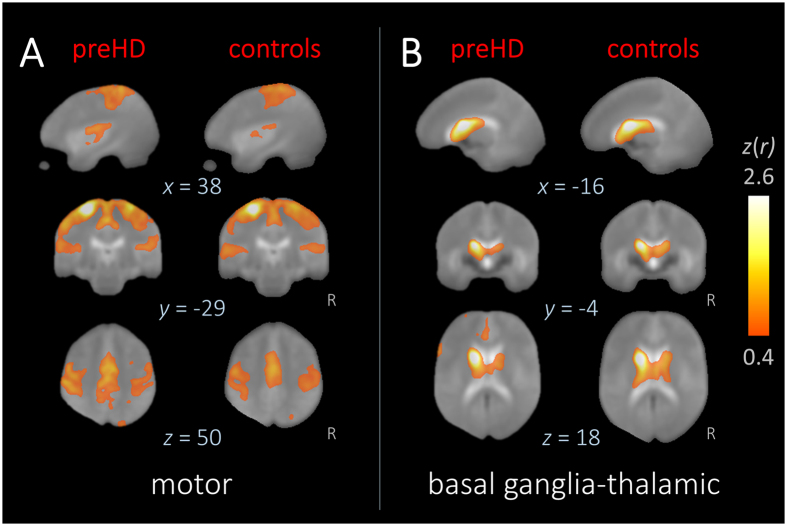
Resting-state functional connectivity networks. Representative orthogonal slices showing *z(r*)-values (thresholded for |*z(r*)| ≥ 0.4) as functional connectivity heat maps in MNI space for (**A**) motor and (**B**) basal ganglia-thalamic networks for far from onset preHD (**left columns**) and controls (**right columns**). Hot colors indicate the strength of the correlation as an indirect measure of functional connectivity. The averaged study-specific echo planar imaging (EPI) is used as background. Voxel-wise group comparisons for each network between far from onset preHD and control subjects revealed no significant differences indicating similar functional networks in both groups.

We next used rs-fMRI to examine brain function. Functional connectivity in a basal ganglia-thalamic and motor networks was similar in far from onset preHD participants compared with controls (Fig. 2), i.e. voxel-wise comparisons revealed no statistically significant differences.

### Structural and functional MRI in early manifest HD

The methods employed here may not have been sensitive enough to detect differences in a relatively small sample compared to a healthy control group. We therefore repeated the structural and functional connectivity analyses in 11 manifest HD patients and controls. This revealed similar differences between groups as reported in a larger cohort of 34 HD patients in our previous study^13^. WBSS of FA-maps revealed FA decrease in HD patients compared to controls in the thalamic region, the internal and external capsule and the corpus callosum (*p* < 0.01, corrected); basal ganglia FA was significantly increased in HD patients compared to controls (*p* < 0.01, corrected), see Supplementary Figure 1A. In the motor network, connectivity of the insular cortex was bilaterally reduced in HD participants compared with controls (*p* < 0.01, corrected).

### Electrophysiology

Data followed a normal distribution, and Levene’s test of unequal variance was not significant for any of the variables. Resting and active motor thresholds were similar in controls (RMT: mean 42.6 (SD 9.3); AMT: 34.27 (9.25)) and far from onset preHD participants (RMT: 48.15 (9.37); AMT: 35.15 (8.61)). All measures of the motor efferent (motor evoked potential size, latency and input-output ratios; cortical silent period) and sensory afferent pathways (N20 latency; N20/P25 amplitude; long-latency reflex latencies and amplitudes) as well as a measure of sensory-motor integration (cortical relay time) were similar in controls and far from onset preHD participants. Table 1 shows the electrophysiological data.

## Discussion

In this study, we assessed sensory-motor brain structure, brain function and clinical measures (UHDRS total motor score and cognition) in a cohort of HD gene mutation carriers far from the predicted clinical onset of HD, and controls. MRI measures of macro- or micro-structure, and brain function measured with rs-fMRI and electrophysiological techniques, as well as clinical measures were similar in the two groups.

For this study, we recruited a cohort of preHD participants who were far from the predicted age at onset and had a disease burden score which was substantially lower than in other preHD study populations (e.g. refs 14, 15, 16, 17). In agreement with many studies, we found no clinically meaningful motor signs, and cognitive task performance was similar to controls. Data from TRACK-HD suggest that clinical measures and task performance, e.g. cognition, can remain stable despite continuous loss of brain tissue^3^. Brain structural changes detected with MRI are a hallmark of manifest HD and precede the onset of clinical signs of HD^18^. This affects grey and white matter; white matter change may precede grey matter loss^3^, and the most prominent changes reported in preHD nearer to onset are those in the striatum^3^. Using VBM and DTI we did not find any abnormalities in our cohort of far from onset preHD. This differs from other reports in preHD that have described both macro- (e.g. GMV^19^) and microstructural change (e.g. DTI markers^20^). Given that the disease burden score in our cohort was substantially lower than in other cohorts (e.g. refs 14, 15, 16, 17) people with far from onset preHD may develop structural changes when they get nearer to manifesting diagnostic signs of clinically manifest HD. This suggests that, at least with the methods we used, the structural changes ascribed to the pathogenesis of HD may not be evident far from motor onset. However, it is possible that more subtle changes may be detectable depending on the methods used without impacting brain function or clinical measures^17^.

We then examined brain function. Using rs-fMRI, intrinsic functional connectivity in two different networks was similar in far from onset preHD and controls. Several recent studies have addressed intrinsic neural function in preHD. Our findings are in accord with a study in a preHD group with a mean disease burden score of about 250 that showed preserved functional connectivity at rest^21^, with some evidence for M1/precuneus decoupling when employing a different analysis technique^22^. Significantly reduced synchronization in the sensory-motor network, particularly in the medial primary motor area (M1) posterior to the supplementary motor area, was detected by another study^23^. Others have reported no difference to controls^24^,^25^. Some studies have also shown correlations between disease burden and functional connectivity^25^. This suggests that changes in functional connectivity may increase with higher disease burden. In our cohort of far from onset preHD the disease burden score was very low (median 202) compared to previous studies in which the mean disease burden score usually ranged between 250 and 300^14^. It is conceivable that functional differences to controls emerge only when the disease burden is higher than in our cohort. As a second method to assess brain function we used electrophysiological methods that probed the sensory-motor circuit. Similar to rs-fMRI, motor efferent and sensory afferent data were similar in controls and far from onset preHD. Previously, shallower motor evoked potential recruitment and reduced plasticity to theta burst TMS had suggested differences in synaptic function in preHD close to motor onset^26^. It is possible that synaptic excitability changes when the biological load in HD increases similar to functional connectivity. This is consistent with a change over time in other electrophysiological measures including SEPs and long-latency reflexes even though this was measured in manifest HD^12^. While this does not exclude the possibility of more subtle abnormalities even in far from onset preHD, consistent with our functional connectivity data these findings are consistent with the idea that only with increasing biological load HD pathogenesis results in a phenotype that can be measured reliably with our methods.

At rest the brain is less challenged than when completing a task. Therefore, task based fMRI could be more sensitive in detecting neural activity differences in far from onset preHD. Indeed, several studies have reported differences to controls in brain activity in preHD with cognitive tasks^6^,^15^,^27^,^28^. This suggests that while intrinsic brain function may be intact, extrinsic challenge may nevertheless reveal subtle changes of brain activity. However, no task-based fMRI study so far has convincingly shown that functional change is detrimental for behavioral performance. On the contrary, intact behavior even in the presence of structural loss over time^3^, together with an increase of task related brain activity in preHD has given rise to the hypothesis of functional compensation, i.e. an increased neural engagement or the recruitment of alternative neural pathways to meet the requirements of a given task. However, data from studies that tested this hypothesis using an a-priori defined model and longitudinal data are scarce^25^.

The investigation of a cohort of far from onset preHD with different modalities is a strength of this study. However, we also acknowledge several limitations. First, the number of far from onset preHD participants was low. Participants have to be recruited through an HD clinic. People carrying the HD mutation may come for predictive testing – and the uptake rate is low – but otherwise have little reason to attend a clinic. The younger they are, and hence the further from disease onset, the less likely they are to attend and, hence, the more difficult they are to recruit for research. However, using the same MRI techniques in 11 manifest HD patients compared to the same controls a pattern clearly emerged of FA and intrinsic functional connectivity differences that was very similar to what we described in a larger cohort of manifest HD patients^13^. This indicates that our methods are sensitive enough to detect differences in small cohorts. The effects in manifest HD may be considerably larger than in far from onset preHD. Hence, smaller effects may not have been detected because of a lack of statistical power. While it would have been desirable to have studied a larger cohort we are nonetheless confident that changes similar in magnitude to our previous study would have been evident. We also have to acknowledge that rs-fMRI is conducted within a poorly controlled experimental environment in which brain activity at rest can be modulated by non-specific factors, such as variation in vigilance. However, since the electrophysiological data agrees with the fMRI data in showing no difference in brain function to controls this supports the notion that brain function at rest in our far from onset preHD cohort was indeed intact. It is nonetheless possible that in far from onset preHD there are more subtle differences to controls that our methods were unable to detect, e.g. changes at the intracellular or synapse level^29^.

Taken together our multi-modal assessment of a cohort of preHD participants far from onset did not reveal any differences to controls in task performance, neural micro- or macro-structure, and brain function measured with rs-fMRI and electrophysiology. The results of this study support the notion that any observable structural or functional change in preHD nearer to onset, and also in manifest HD, at least when using comparable techniques such as in this study, most likely reflects an ongoing neurodegenerative process.

## Methods

In preHD, as far as possible from motor onset, we investigated sensory-motor clinical measures, and used neuroimaging and electrophysiological techniques to investigate macrostructure, microstructure, and network function. The statistical analysis was adapted to each of the modalities using parametric or non-parametric statistics depending on data distribution and assuming unequal variance given the differences in the number of preHD cases and controls. Unless specifically mentioned, and adjusted for, age had no significant influence on any of the variables.

### Participants

Twelve pre-symptomatic carriers (preHD) of the HD-gene mutation and 22 age and sex matched healthy controls were recruited from the Huntington’s disease clinic at Ulm University Hospital between 2012 and 2014. Far from onset preHD participants were eligible when they had a diagnostic confidence score of 2 or less on the motor Unified Huntington’s disease ratings scale (UHDRS)^30^, a molecular genetic diagnosis of a CAG repeat expansion (≥36) in the *HTT* gene^30^ and a disease burden score of less than 250^8^. The UHDRS was used to determine total motor score and cognitive scores (verbal fluency, Stroop word reading, Stroop colour naming, Stroop interference, and Symbol Digit Modalities Test). Controls were healthy partners of HD patients or volunteers. Participants with another neurological disease or psychiatric disorder according to DSM-IV-TR criteria, a medical condition, substance abuse or dependence were excluded. None of the controls were on medication. All participants were native German speakers. Table 2 summarizes demographic and clinical details for all participants.

The study protocol was approved by the local ethics committee of Ulm University, Germany. Participants gave their written informed consent, and all methods were used and experiments performed, in accordance with the ethical standards laid down in the 1964 Declaration of Helsinki and its later amendments.

### MRI data acquisition

All participants except one far from onset preHD participant underwent whole-brain MRI using a 3 Tesla MRI scanner (Magnetom Allegra, Siemens Medical, Erlangen, Germany) including DTI, rs-fMRI and T_1_-weighted 3-D sequences.

The DTI protocol consisted of 49 gradient directions (in-plane pixel size 2.2 mm × 2.2 mm, slice thickness 2.2 mm (no gap), TE 85 ms, TR 7600 ms, b = 1000 s/mm^2^) including one b = 0 gradient direction (52 slices, 96 × 128 pixels).

Rs-fMRI was performed using a BOLD sensitive T2* -weighted echo planar imaging sequence with an overall acquisition time of 6:00 minutes (180 volumes, 36 slices, 64 × 64 pixels, slice thickness 3.0 mm, in-plane pixel size 3.0 mm × 3.0 mm, TE 30 ms, TR 2000 ms). Participants were advised to stay motionless and relaxed with their eyes closed but to remain awake throughout the rs-fMRI sequence.

High-resolution T1 weighted 3-D scans were acquired using magnetization prepared rapid acquisition gradient echo (MPRAGE) sequence (192 sagittal slices, echo distance 10.8 ms, flip angle 7°, pixel size 1.0 mm × 1.0 mm × 1.0 mm, TE 432 ms, TR 2500 ms).

### DTI and rs-fMRI data analysis

#### Overview

Functional and diffusion weighted imaging data were analyzed using the *Tensor Imaging and Fiber Tracking* (TIFT) software package^31^. Warping for DTI and rs-fMRI, to Montreal Neurological Institute (MNI) stereotaxic standard space was applied by use of a study-specific template ((*b* = 0)/EPI) together with landmarks defined on the individual DTI and rs-fMRI imaging data, respectively^32^. The data analysis pipeline for DTI and rs-fMRI data followed the same procedure as published in our previous study on manifest HD patients^13^. The data were resampled from the default scanner resolution to a cubic 1 mm grid of a 256 × 256 × 256 matrix by means of a nonparametric *k*-nearest neighbor regression approach using the average voxel intensity of the k-nearest neighbor voxels weighted by the inverse of their distance.

#### DTI data analysis

For DTI data, additionally to the MNI normalization, a normalization to fractional anisotropy (FA) maps was performed preserving directional information. Maps of FA, radial diffusivity (RD), and axial diffusivity (AD) were computed, and a Gaussian smoothing filter of 8 mm full-width at half maximum (FWHM) was applied to the individual normalized FA/RD/AD-maps using a standardized procedure as previously described in detail^33^. Next, fiber tracking was performed with a seed-to-target approach in order to study the structure of the corticospinal tract (CST) and the thalamus to somatosensory cortex connection (thalamocortical pathway – TCP). For this purpose, spherical start and target regions of interest were defined for the (1) CST start regions at (MNI x = ±6/y = −38/z = −29, r = 5 mm) and target regions (MNI x = ±20/y = −29/z = 59, r = 20 mm), and for the (2) TCP start regions at (MNI x = ±21/y = −19/z = 10, r = 5 mm) and target regions at (MNI x = ±21/y = −45/z = 47, r = 20 mm). Voxels with an FA value of above a threshold of 0.2 were considered. For the fiber tracking technique, a modified deterministic streamline tracking approach was used that takes the directional information of FTs in the vicinity into account. For these pathways, the FT algorithm was not compromised by the presence of crossing fibers.

#### rs-fMRI data analysis

Preprocessing of rs-fMRI data followed conventional practice^34^. Briefly, this included (1) resampling to a cubic 1 mm grid, (2) rigid body correction of head movement within runs, (3) deformation to MNI stereotaxic space using a study-specific template together with landmarks defined on the individual imaging data, (4) spatial smoothing (7 mm full-width at half maximum Gaussian blur filter in each direction), (5) voxel-wise removal of linear trends and zero-meaning, and (6) temporal bandpass filtering (0.01–0.08 Hz). The rs-fMRI deformation procedures additionally considered possible gray-matter atrophy as a confounding factor on functional connectivity analysis. Two large-scale correlation maps, i.e. intrinsic connectivity networks (ICN), were computed according to previously defined well-recognized networks^34^ using the seed-based approach, i.e., (1) motor (voxel-seeds: motor cortex; MNI coordinates: x = ±27/y = −37/z = 68) and (2) basal ganglia-thalamic (caudate nucleus; x = ±18/y = −2/z = 20). The extracted time-courses of the seed-voxels were correlated with the time series of all other voxels across the whole brain and the resulting correlation maps (*r*-values) were voxel-wise Fisher’s *r-* to-*z* transformed.

### Statistical analysis of DTI and rs-fMRI data

Statistical interference between the FFO-HD group and controls were tested using the two-sided parametric unpaired Student’s *t-*test for unequal variances for both whole brain-based voxel-wise spatial statistical (WBSS) and voxel-wise comparison of connectivity z(r) maps; *p* < 0.05 indicated statistical significance. The resulting *p*-values were corrected for multiple comparisons using the false discovery rate (FDR) approach at 5% level. Further correction for multiple comparisons for reduction of the alpha error was performed by a parametric correlation-based clustering procedure that discarded isolated clusters not exceeding the minimum size of 343 voxels at cubic 1 mm resolution. The resulting FA/RD/AD from fiber tracking of the CST and TCP were compared between FFO-HD and control subjects using the non-parametric Wilcoxon-Mann-Whitney-*U*-test. Voxels with FA values below 0.2 were not considered for statistical analyses as cortical grey matter shows FA values up to 0.2.

### Voxel-based morphometry analysis

We used the VBM8 toolbox (http://dbm.neuro.uni-jena.de/vbm8/) running under the Statistical Parametric Mapping software package (version 8, SPM8; http://www.fil.ion.ucl.ac.uk/spm). In VBM8, the segmentation algorithm is based on an adaptive “Maximum A Posterior” (MAP) technique^35^. During MAP estimation local parameter variations were modeled as varying spatial functions, accounting for intensity inhomogeneity and other local intensity variations. Each participant’s original T1 image was spatially normalized and segmented into gray and white matter and cerebrospinal fluid based on MAP estimation. Segmentation was followed by partial volume estimation, data denoising based on a spatially adaptive non-local means filter^36^ and application of Markov Random Fields. After data preprocessing, modulated normalized gray and white matter volumes (GMV/WMV) were smoothed using an 8 mm Full Width at Half Maximum (FWHM) Gaussian kernel prior to 2^nd^ level between-group analyses. GMV and WMV differences between the groups were calculated using two-sample t-test models adjusted for age and gender. An absolute threshold of 0.2 was used to prevent effects located at tissue border regions. A threshold of p < 0.001, uncorrected at the voxel level, p < 0.05 corrected for spatial extent was chosen.

Region-of-interest (ROI)-based analyses were used to investigate caudate and putamen GMV differences in far from onset preHD compared to controls. For this purpose, we first entered GMV images for both groups into a one-sample *t*-test model adjusted for age and sex and restricted these analyses to bilateral caudate and putamen volumes as defined by the Automatic Anatomical Labeling (AAL) atlas^37^. Subsequently, we extracted cluster-wise mean GMV parameter estimates representing the extent of caudate and putamen GMV within a group. These values were the processed offline using t-tests (nominal p < 0.05, Bonferroni corrected p-value < 0.0125), as provided by the Statistica software package (Version 10, http://www.statsoft.de/).

### DTI and rs-fMRI data in early manifest HD

From our previous study on structural and functional connectivity analysis in early manifest HD patients^13^, we randomly selected 11 HD patients (age 35–60 years, 7 male, disease duration 1–14 years, diseases burden 263–483, CAG repeats 41–46, UHDRS motor score 2–37) who were age-matched (Mann-Whitney-*U p* = 0.130) with the control cohort (*N* = 22) used in this study. We compared functional connectivity network patterns from rs-fMRI data and WBSS from DTI data between these HD cases (*N* = 11) and controls using the identical data analysis stream as described above. We aimed at investigating whether the previously reported results in ref. 13 could be replicated in a subgroup of 11 (out of 34) manifest HD patients, i.e. in a cohort with the same number of participants as used in this study.

### Electrophysiology

All far from onset preHD participants and 13 controls took part in the electrophysiology experiments. Somatosensory evoked potentials (SSEP) were recorded following median nerve stimulation using routine techniques^38^. In brief, SSEPs were recorded with a silver/silver-chloride disk electrode over the somatosensory cortex (2 cm posterior of C3 in the international EEG 10–20 system) referenced against Fz. Stimulation at 3 Hz was given at 150% of the motor threshold; recordings from a total of 300 stimuli were averaged, followed by N20 latency and N20/P25 amplitude determination. Latencies were corrected for arm length measured between the cathode of the stimulation electrodes and the seventh vertebra. Surface electromyograms (EMG) were recorded from the right abductor pollicis brevis (APB) muscle using silver/silver-chloride disc surface electrodes (1 cm diameter) in a belly tendon montage. The EMG signal was amplified with a universal amplifier (Toennies-Jaeger, Höchberg, Germany) and bandpass filtered (20 Hz < *f* < 2 kHz). Data (sampling rate 5 kHz) were digitised for off-line analysis using A/D data acquisition board (DAP 4200a, Microstar Laboratories, Bellevue, WA), controlled by custom software in Dasylab (V 11, National Instruments, measX GmbH & Co KG, Moenchengladbach, Germany).

Transcranial magnetic stimulation (TMS) was done as previously described using established techniques^39^. The protocol included APB hot-spot and motor threshold (active and rest) determination, motor evoked potential latencies and amplitudes, input/output curves at rest (110%, 130%, 150% resting motor threshold) and with pre-activation (125%, 150%, 175% active motor threshold), and silent period determination. Long-latency reflexes were elicited in the contracted right abductor pollicis brevis muscle by electrical stimulation of the median nerve at the wrist as described previously^38^.

For analysis, data were first examined for normal distribution using the Kolmogorov-Smirnov test and for normal variance using Levene’s test. To examine group differences between HD gene carriers far from onset and healthy control subjects and to control for multiple testing, we used a one-way analysis of variance (ANOVA) with the between subjects factor Group. As independent variables, N20 and long-latency reflex amplitudes and latencies, as well as active and rest MEP areas, and silent period duration were entered into the analysis. Statistical significance levels were set to p = 0.05. All statistical analyses were performed using SPSS 20 for Windows software package.

## Additional Information

**How to cite this article**: Gorges, M. *et al*. Intact sensory-motor network structure and function in far from onset premanifest Huntington’s disease. *Sci. Rep.* **7**, 43841; doi: 10.1038/srep43841 (2017).

**Publisher's note:** Springer Nature remains neutral with regard to jurisdictional claims in published maps and institutional affiliations.

## Supplementary Material

Supplementary Dataset 1

## Figures and Tables

**Table 1:** Electrophysiology.

	preHD	Controls
MEP Latency	22.76 (1.67)	22.84 (1.47)
MEP Area at 110% RMT	2.63 (1.65)	2.51 (2.91)
MEP Area at 130% RMT	7.66 (6.61)	5.00 (4.51)
MEP Area at 150% RMT	13.08 (11.50)	8.68 (6.40)
MEP Area at 125% AMT	10.28 (4.14)	11.41 (6.27)
MEP Area at 150% AMT	18.74 (8.69)	20.29 (10.59)
MEP Area at 175% AMT	25.14 (10.02)	25.96 (13.83)
CSP at 125 AMT	94.92 (33.11)	93.46 (34.03)
Ratio MEP/CSP at 125% AMT	0.12 (0.05)	0.14 (0.10)
CSP at 150% AMT	141.17 (39.90)	162.15 (49.65)
Ratio MEP/CSP at 150% AMT	0.14 (0.06)	0.14 (0.10)
CSP at 175% AMT	178.67 (37.75)	162.15 (49.64)
Ratio MEP/CSP at 175% AMT	0.14 (0.05)	0.17 (0.08)
N20 Amplitude	3.23 (1.72)	4.22 (1.61)
N20 Latency	19.20 (2.06)	19.92 (1.76)
LLR-I Latency	41.68 (5.14)	42.19 (3.70)
LLR-I Amplitude	0.10 (0.09)	0.09 (0.06)
LLR-II Latency	49.95 (1.69)	50.53 (4.31)
LLR-II Amplitude	0.07 (0.04)	0.08 (0.06)
Cortical Relay Time	8.08 (2.37)	8.23 (4.90)

Means and standard deviations of electrophysiological data for preHD (n = 12) and healthy controls (n = 13). Abbreviations: LLR: long latency reflex; MEP: motor evoked potential; RMT: resting motor threshold; AMT: active motor threshold; CSP: cortical silent period.

**Table 2:** Demographics and clinical characteristics.

	Far from onset preHD (*N* = 12)	Healthy controls (*N* = 22)	*p*-value
Sex (male:female)	**3:9**	**15:7**	0.030^a^
Age/y	**36** (32–46), 27–62	**45** (39–49), 25–51	0.287^b^
UHDRS^b^ total motor score	**2** (1–3), 0–9		
Years of education	**14** (12–14), 9–17	**17** (14–18), 12–19	0.047^b^
CAGn repeat size	**40** (40–42), 39–44	n/a	n/a
Disease-burden score^d^	**202** (169–222), 102–233	n/a	n/a
Predicted years to onset^e^	**23** (19–29), 14–43	n/a	n/a
Verbal fluency	**42** (37–50), 30–54	**41** (33–50), 25–52	0.851^b^
Stroop color	**80** (62–91), 35–100	**79** (69–88), 48–96	0.808^b^
Stroop interference	**55** (44–64), 18–106	**49** (44–54), 34–65	0.359^b^
Stroop read	**97** (78–114), 43–128	**100** (92–134), 74–150	0.201^b^
SDMT^f^	**56** (46–67), 25–79	**53** (50–60), 35–85	0.940^b^

Data are shown as median (interquartile range), min–max. ^a^Fisher’s exact test; ^b^Mann-Whitney-*U*-test; ^c^Unified Huntington’s Disease Rating Scale; ^d^Disease burden score [age × (CAGn-35.5)]; ^e^Estimated disease onset at a 60% certainty level according to refs 9 and 40; ^f^Symbol Digit Modalities Test; NA, not applicable.
