# Supplementary material for: Intact sensory-motor network structure and function in far from onset premanifest Huntington’s disease
Source: Sci Rep. 2017 Mar 7;7:43841. doi: 10.1038/srep43841 (PMC5339687; doi:10.1038/srep43841)
Supplement: Supplementary Dataset 1 [file srep43841-s1.pdf]

Intact sensory-motor network structure and function in far from onset premanifest Huntington's disease

Authors: Martin Gorges*[1], Hans-Peter Müller*[1], Isabella Maria Sophie Mayer[1], Gesa Sophie Grupe[1], Thomas Kammer[2], Georg Grön[2], Jan Kassubek[1], G. Bernhard Landwehrmeyer[1], Robert Christian Wolf[3], Michael Orth[1]

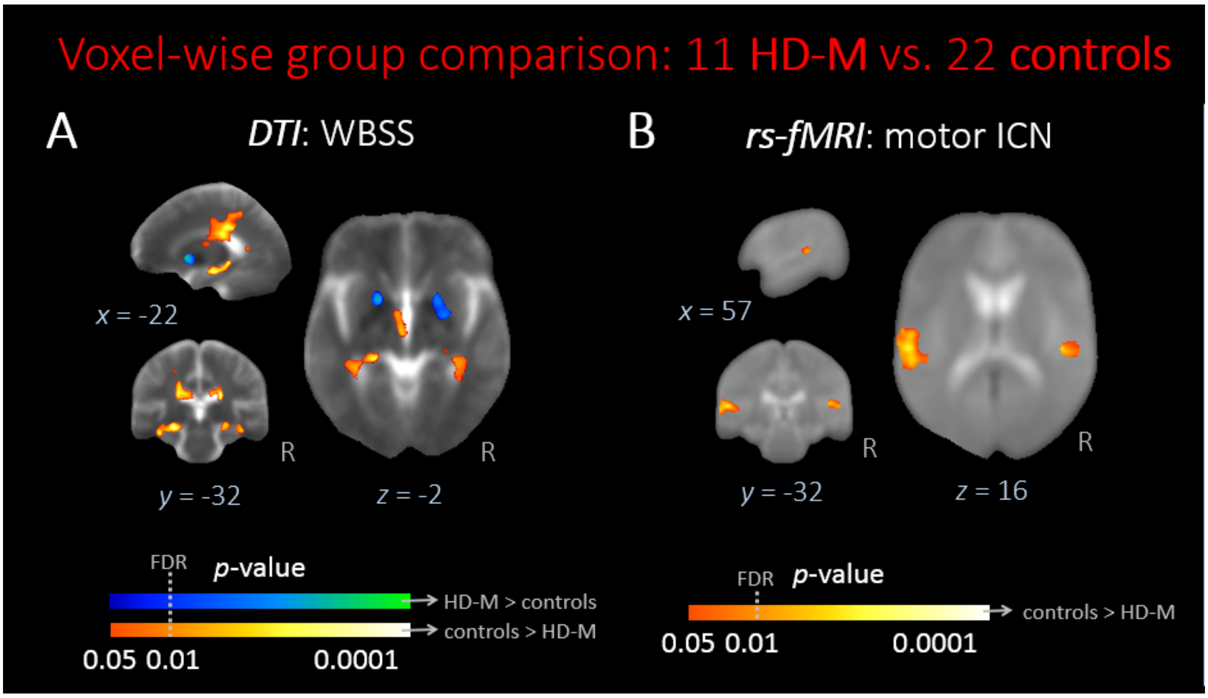

Supplementary Figure 1: Replication of functional and structural analyses in a cohort of 11 manifest HD patients (HD-M) compared with controls as reported previously[16]. (**A**) Diffusion tensor imaging (DTI)-based voxel-wise spatial statistics and (**B**) resting-state (rs-) fMRI analysis of the motor intrinsic functional connectivity network (ICN) in manifest HD patients (*N*=11) compared with controls (*N*=22) depict heat maps of the most representative orthogonal slices in MNI coordinates. (A) Fractional anisotropy (FA-) maps show decreased (hot colors) and increased (cool colors) FA values in HD patients compared with controls. (B) Heat maps showing clusters indicate significantly decreased (hot colors, $p<0.05$, FDR and cluster-wise corrected) motor network functional connectivity in HD patients compared with controls. (A, B).
